# Hybrid de novo genome assembly of the Chinese herbal fleabane *Erigeron breviscapus*

**DOI:** 10.1093/gigascience/gix028

**Published:** 2017-04-18

**Authors:** Jing Yang, Guanghui Zhang, Jing Zhang, Hui Liu, Wei Chen, Xiao Wang, Yahe Li, Yang Dong, Shengchao Yang

**Affiliations:** 1 Biological Big Data College, Yunnan Agricultural University, Kunming 650201, China; 2 National-Local Joint Engineering Research Center on Germplasm Utilization and Innovation of Chinese Medicinal Materials in Southwest China, Yunnan Agricultural University, Kunming 650201, China; 3 NOWBIO Technology Co. Ltd, Kunming 650202, China; 4 State Key Laboratory of Genetic Resources and Evolution, Kunming Institute of Zoology, Chinese Academy of Sciences, Kunming 650223, China; 5 University of Chinese Academy of Sciences, Beijing 100049, China; 6 Yunnan Research Institute for Local Plateau Agriculture and Industry, Kunming 650201, China; 7 Longjin Pharmaceutical Co. Ltd, Kunming 650228, China; 8 College of Life Science, Kunming University of Science and Technology, Kunming 650500, China

**Keywords:** *Erigeron breviscapus*, Illumina sequencing, PacBio sequencing

## Abstract

**Background:** The plants in the *Erigeron* genus of the Compositae (Asteraceae) family are commonly called fleabanes, possibly due to the belief that certain chemicals in these plants repel fleas. In the traditional Chinese medicine, *Erigeron breviscapus*, which is native to China, was widely used in the treatment of cerebrovascular disease. A handful of bioactive compounds, including scutellarin, 3,5-dicaffeoylquinic acid, and 3,4-dicaffeoylquinic acid, have been isolated from the plant. With the purpose of finding novel medicinal compounds and understanding their biosynthetic pathways, we propose to sequence the genome of *E. breviscapus*. **Findings:** We assembled the highly heterozygous *E. breviscapus* genome using a combination of PacBio single-molecular real-time sequencing and next-generation sequencing methods on the Illumina HiSeq platform. The final draft genome is approximately 1.2 Gb, with contig and scaffold N50 sizes of 18.8 kb and 31.5 kb, respectively. Further analyses predicted 37 504 protein-coding genes in the *E. breviscapus* genome and 8172 shared gene families among Compositae species. **Conclusions:** The *E. breviscapus* genome provides a valuable resource for the investigation of novel bioactive compounds in this Chinese herb.

## Background

**Figure 1:**
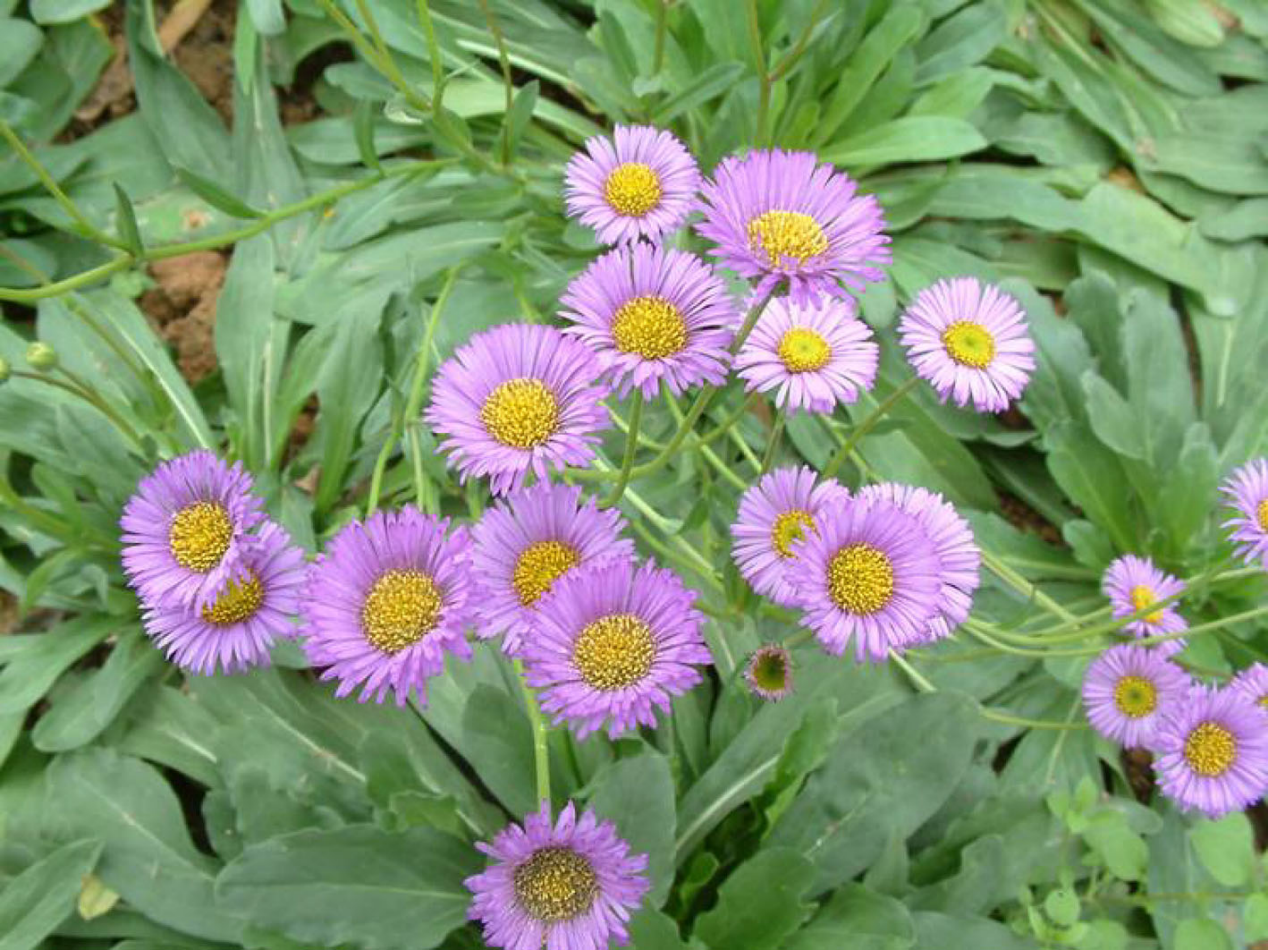
Example of the *E. breviscapus* (image from Shengchao Yang).

*Erigeron breviscapus* (also known as *dengzhanhua* in Chinese) is a perennial flower in the *Erigeron* genus of the Compositae (Asteraceae) family. Its flower head is comprised of yellow disk florets and multiple surrounding blue to purple ray florets (Fig. 1). This species is endemic to Southwestern China, and it grows in mid-altitude mountains, subalpine open slopes, grasslands, and forest margins from 1000 m to 3500 m [1, 2]. In traditional Chinese medicine, *E. breviscapus* is believed to improve blood circulation and ameliorate platelet coagulation [3, 4]. Since the 1980s, herbal extracts and bioactive compounds from *E. breviscapus* have been widely used for the treatment of cerebral embolism and its complications, cerebral thrombosis, coronary heart disease, angina pectoris, acute renal failure, and nephritic syndrome [5]. At present, more than 1000 tons of dry *E. breviscapus* are collected and used in the pharmaceutical industry each year, greatly exhausting the wild resources of this species [6, 7]. In this study, we report the draft genome assembly of *E. breviscapus*. Because of the high heterozygosity of the *E. breviscapus* genome, we adopted both Illumina sequencing and PacBio single-molecular real-time sequencing in the assembly procedure.

## Data Description

### Whole-genome shotgun sequencing of *E. breviscapus* on Illumina platform

*E. breviscapus* seedlings were provided by Longjin Pharmaceutical Co., Ltd., and maintained in a greenhouse at the Yunnan Agricultural University. Genomic DNA was extracted from the leaf tissues of a single *E. breviscapus* plant using the GenElute^TM^ Plant Genomic DNA Miniprep Kit (Sigma-Aldrich, USA). Paired-end libraries with insert sizes ranging from 150 bp to 800 bp were constructed using NEBNext Ultra II DNA Library Prep Kit for Illumina (NEB, USA), and mate pair libraries with insert sizes from 2 kb to 20 kb were constructed using Illumina Nextera Mate Pair Library Preparation Kit (Illumina, USA). All constructed libraries were sequenced on a HiSeq 2500 platform (Illumina, USA) using either a PE-100 or PE-90 module (Additional File 1: Table S1). In total, about ∼413.4 Gb of raw data were generated on the Illumina platform. All reads were preprocessed for quality control and filtered using our in-house Perl script. The raw data were initially filtered by removing reads with more than 10% N or more than 40 bp low-quality bases. Next, redundant reads resulting in duplicate base calls were filtered at a threshold of Euclidean distance ≤3 and mismatch rate of ≤0.1. Only one copy of any duplicated paired-end reads was retained. Finally, both read 1 and read 2 were removed if they contained an adapter ≥10 bp with a mismatch rate ≤0.1. This process yielded ∼275.1 Gb of clean data for the de novo assembly of the *E. breviscapus* genome (Additional File 1: Table S1).

### Single-molecule real-time sequencing of long reads on PacBio platform

Single-molecule real-time (SMRT) sequencing of long reads on a PacBio RS II platform (Pacific Biosciences, USA) was used to assist the subsequent de novo genome assembly process [8]. In brief, 40 μg of sheared DNA was used to construct 26 SMRT Cell libraries with an insert size of 17 kb. These libraries were sequenced in 105 SMRT DNA sequencing cells using the P6 polymerase/C4 chemistry combination and a data collection time of 240 minutes per cell. The sequencing produced about 62.4 Gb of clean data, consisting of 6 802 553 reads with an average read length of 9175 bp (Additional File 1: Table S1).

### Estimation of the *E. breviscapus* genome size

The genome size of *E. breviscapus* was estimated by flow cytometry using *Oryza sativa* Nipponbare as internal standard and propidium iodide as the stain. The result showed that the genome size of *E. breviscapus* was approximately 1.52 Gb (Additional File 1: Figure S1).

### Estimation of the *E. breviscapus* genome heterozygosity using *k*-mer analysis

Quality-filtered reads from the Illumina platform were subjected to 23-mer frequency distribution analysis with Jellyfish (v. 2.2.5) [9]. Analysis parameters were set at -k 23, and the final result was plotted as a frequency graph (Additional File 1: Figure S2). Two distinctive modes were observed from the distribution curve: the lower peak at a depth of 57 reflected the high heterozygosity of the *E. breviscapus* genome.

### Hybrid de novo genome assembly of *E. breviscapus*

**Figure 2:**
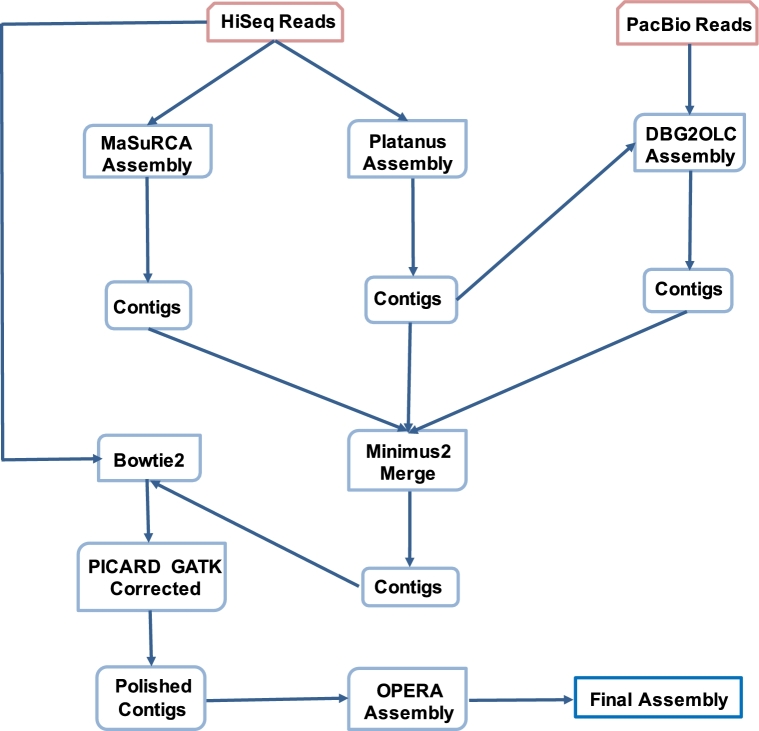
Assembly pipeline for the *E. breviscapus* genome.

A hybrid genome assembly pipeline was used to overcome challenges posed by the heterozygous *E. breviscapus* genome (Fig. 2). HiSeq reads were first assembled using MaSuRCA (v. 3.1.3) [10] with default parameters, and also using Platanus (v. 1.2.1) [11] with parameters “-m 500 -k 43 -s 5 -d 0.3 -u 0.15 -c 3,” resulting in two contig assemblies. The Platanus-generated contigs, together with PacBio reads, were used to generate a third contig assembly by DBG2OLC with default parameters [12]. The three different contig assemblies were merged together by Minimus2 Amos (v. 3.1.0) using default parameters [13]. To eliminate possible errors of the merged contig assembly: (i) Bowtie2 (v. 2.1.0) [14] was used to align Hiseq reads back to this contig assembly. The resultant SAM file was changed into a BAM file by SAMtools (v. 0.1.19-44428cd) [15] with the command “samtools view -bS.” (ii) Redundant sequences resulting from polymerase chain reaction amplification were removed with PICARD (v. 1.134; http://picard.sourceforge.net) using the command “MarkDuplicates.” (iii) The single nucleotide polymorphisms and indels were called from short-read alignments and used to correct the contigs by GATK (v. 3.4-0-g7e26428) [16, 17] with the command “HaplotypeCaller” and “FastaAlternateReferenceMaker,” respectively. The final polished contig number was 464 088 with N50 of 18.8 kb. Polished contigs were then used to build scaffolds using OPERA (v. 2.0.1) [18] with a *k*-mer of 39. This process yielded a final draft *E. breviscapus* genome of 1.2 Gb, with a contig N50 size of 18.8 kb and a scaffold N50 size of 31.5 kb (Additional File 1: Table S2).

### Evaluation of the completeness of the *E. breviscapus* genome assembly

We evaluated the completeness of the final assembly using the Core Eukaryotic Genes Mapping Approach (CEGMA; v. 2.5) [19] with a set of 248 ultra-conserved core eukaryotic genes and Benchmarking Universal Single-Copy Orthologs (BUSCO; v. 2.0) [20] with the Embryophyta gene set. CEGMA assessment showed that our assembly captured 240 (96.9%) of the 248 ultra-conserved core eukaryotic genes, of which 217 (87.5%) were complete (Table 1). BUSCO analysis showed that 80.6% and 6.3% of the 1440 expected embryophytic genes were identified as complete and fragmented, respectively (Table 2).

**Table 1:** Statistics of the completeness of the hybrid de novo assembly genome of *E. breviscapus* by CEGMA.

Group	Protein Num^a^	Completeness (%)^b^	Total Num^c^	Average Num^d^	Ortholog (%)^e^
Complete	217	87.50	633	2.92	82.95
Group 1	58	87.88	158	2.72	77.59
Group 2	49	87.50	126	2.57	77.55
Group 3	53	86.89	171	3.23	96.23
Group 4	57	87.69	178	3.12	80.70
Partial	240	96.77	856	3.57	89.58
Group 1	63	95.45	206	3.27	85.71
Group 2	55	98.21	185	3.36	83.64
Group 3	59	96.72	232	3.93	98.31
Group 4	63	96.92	233	3.70	90.48

^a^Protein Num.: Number of 248 ultra-conserved core eukaryotic genes (CEGs) present in the *E. breviscapus* genome.

^b^Completeness (%): Percentage of 248 ultra-conserved CEGs present in the *E. breviscapus* genome.

^c^Total Num.: Total number of CEGs including putative orthologs present in the *E. breviscapus* genome.

^d^Average Num: Average number of orthologs per CEG.

^e^Ortholog (%): Percentage of detected CEGs that have more than one ortholog.

**Table 2:** Statistics of the completeness of the hybrid de novo assembly genome of *E. breviscapus* by BUSCO.

BUSCO benchmark	Number	Percentage (%)
Total BUSCO groups searched	1440	–
Complete BUSCOs	1161	80.63
Complete and single-copy BUSCOs	635	44.10
Complete and duplicated BUSCOs	526	36.53
Fragmented BUSCOs	90	6.25
Missing BUSCOs	189	13.13

### Transcriptome sequencing

Total RNA was extracted from the leaf, root, stem, and flower tissues of a cultivated *E. breviscapus* individual using Qiagen RNeasy Plant Mini Kits. Additional RNA samples of the leaf tissues were acquired from six more cultivated individuals and five wild individuals (Additional File 1: Table S3). All cultivated samples were acquired from the greenhouse, and all wild samples were collected from Dali, Yunnan Province. Total RNA-seq libraries were prepared using TruSeq RNA Library Preparation Kit, v. 2 (Illumina, CA, USA), according to the manufacturer's instructions and subsequently sequenced on the HiSeq 2500 platform. In total, about 1.1 billion RNA-seq reads were obtained, representing ∼117.6 Gb of raw data. We aligned all the RNA-seq reads back to the *E. breviscapus* genome assembly using TopHat (v. 2.0.10) [21] with default parameters (Additional File 1: Table S3). The percentage of aligned reads ranged from 60.6% for the root to 80.9% for the leaf. We also calculated that 177 886 122 RNA-seq reads were mapped outside of the annotated regions using HTSeq (v. 0.6.1p1) [22] with the command “htseq-count –a 0.” The FPKM value was calculated for each protein-coding gene by Cufflinks (v. 2.1.1) using default parameters. FPKM >0.05 was used as the cutoff value to identify expressed genes.

### Repeat annotation of the *E. breviscapus* genome assembly

The *E. breviscapus* genome was searched for tandem repeats using the Tandem Repeat Finder (v. 4.07b) [23]. RepeatMasker (v. 3.3.0) and RepeatProteinMasker [24] were used against Repbase library (v. 18.07) [25] to identify known transposable element repeats. De novo evolved transposable element annotation was performed using RepeatModeler (v. 1.0.8) [24] and LTR FINDER (v. 1.0.5) [26]. The combined results show that the total length of repeated sequences is about 664.2 Mb, accounting for ∼54.58% of the *E. breviscapus* genome assembly (Additional File 1: Tables S4 and S5).

### Gene prediction

We used multiple methods to annotate protein-coding genes in the *E. breviscapus* genome, including homology-based predictions, de novo predictions, and transcriptome-based predictions. For homology-based predictions, protein sequences of *Arabidopsis thaliana, Fragaria vesca, Malus domestica, Oryza sativa, Prunus persica*, and *Vitis vinifera* were obtained from Phytozome, v. 9.1 (http://www.phytozome.net/), *Pyrus communis* from Genome Database for Rosaceae (https://www.rosaceae.org), and *Prunus mume* from the National Center for Biotechnology Information (ftp://ftp.ncbi.nih.gov/genomes/Prunus_mume). First, query sequences were subjected to TBLASTN analysis with a cutoff E-value of 1e^−5^. BLAST hits corresponding to reference proteins were concatenated by Solar (v. 0.9.6; The Beijing Genomics Institute [BGI] development) [27] after low-quality records were removed. The genomic sequence of each reference protein was extended upstream and downstream by 2000 bp to represent a protein-coding region. GeneWise (v. 2.2.0) [28] was used to predict the gene structure contained in each protein region. For de novo predictions, AUGUSTUS (v. 2.5.5) [29], GENSCAN (v. 1.0) [30], SNAP (released 29 November 2013) [31], and glimmerHMM (v. 3.0.2) [32] analyses were performed on the repeat-masked genome, with parameters trained from *A. thaliana*. For transcriptome-based predictions, RNA-seq data from the leaves of six cultivated individuals were used for gene annotation, processed by Tophat and Cufflinks. The homology, de novo–, and transcriptomic-based predicted gene sets were merged to form a comprehensive and non-redundant reference gene set using Evidence Modeler (released 25 June 2012) [33]. We filtered gene models using our in-house Perl script in by the following criteria: (i) genes with incomplete open reading frames, (ii) small genes with a protein-coding region <150 bp, (iii) stop codons present in the middle of the gene, (iv) genes containing only one exon, and not supported by transcriptome-based evidence. Our analysis indicates that the *E. breviscapus* genome contains 37 504 protein-coding genes with an average coding DNA sequence length of 1034 bp (Additional File 1: Table S6).

### Non-coding RNA annotation

tRNAscan-SE (v. 1.3.1) [34] with default parameters for eukaryotes was used for tRNA annotation. Homology-based rRNA annotation was performed by mapping plant rRNAs to the *E. breviscapus* genome using BLASTN with parameters of “E-value = 1e^−5^.” miRNA and snRNA genes were predicted by INFERNAL (v. 1.1) [35] using the Rfam database (release 11.0) [36]. The final results include 504 miRNAs, 751 tRNAs, 159 rRNAs, and 385 snRNAs (Additional File 1: Table S7).

### Gene family clustering analysis

**Figure 3:**
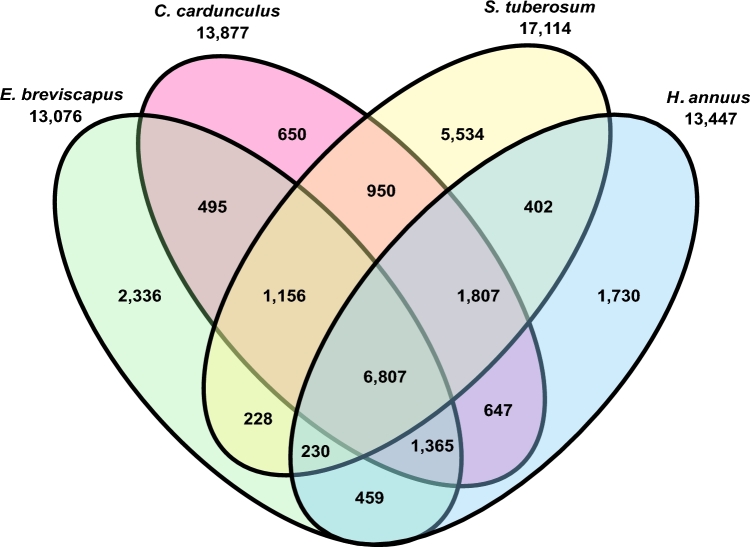
Venn diagram showing unique and shared gene families among four sequenced dicotyledonous species.

To identify and estimate the number of potential orthologous gene families between *E. breviscapus, Helianthus annuus, Cynara cardunculus, Solanum tuberosum, Solanum lycopersicum, V. vinifera*, and *O. sativa*, we applied the OrthoMCL (v. 2.0.9) pipeline [37] using standard settings (BLASTP E-value < 1e^−5^) to compute the all-against-all similarities. Gene sequences from *S. tuberosum, S. lycopersicum, V. vinifera*, and *O. sativa* were downloaded from Phytozome, v. 11.0. Gene sequences from *H. annuus* and *C. cardunculus* were downloaded from the Sunflower Genome Database (http://www.sunflowergenome.org) and Globe artichoke GBrowse (http://gviewer.gc.ucdavis.edu/cgi-bin/gbrowse/Artichoke_v1_1), respectively. Among the total 13 076 *E. breviscapus* gene families, 2336 (17.9%) appear to be lineage specific. There are 8172 (41.8%) gene families shared among Compositae species including *E. breviscapus, H. annuus*, and *C. cardunculus*. In addition, *E. breviscapus* shared 8421 (64.4%) gene families with *S. tuberosum* (Fig. 3).

### Phylogenetic tree construction and divergence time estimation

All 389 single-copy orthologous genes identified in the gene family clustering analysis from the *S. lycopersicum, V. vinifera, O. sativa, E. breviscapus, H. annuus, C. cardunculus*, and *S. tuberosum* were used to construct a phylogenetic tree. Orthologous genes from the seven species were aligned using MUSCLE (v3.8.31) with default settings [38] for each gene. Four-fold degenerate sites were extracted from each gene and concatenated into a ‘‘super gene’’ for each species. PhyML (v. 3.0) [39] was used to reconstruct phylogenetic trees between species. We implemented a Monte Carlo Markov chain (MCMC) algorithm for the estimation of divergence times using the program MCMCtree from the PAML package [40]. The result showed that *E. breviscapus* shared a closer phylogenetic relationship with *H. annuus* than *C. cardunculus* in the Compositae family (Additional File 1: Figure S3). The estimated divergence time was 29.7 million years ago between *E. breviscapus* and *H. annuus* (Additional File 1: Figure S4).

### Expansion and contraction of gene families

CAFE (v. 2.1) [41] is a tool for analyzing the evolution of gene family size based on the stochastic birth and death model. With the calculated phylogeny and the divergence time, this software was applied to identify gene families that had undergone expansion and/or contraction in *S. lycopersicum, V. vinifera, O. sativa, E. breviscapus, H. annuus, C. cardunculus*, and *S. tuberosum* with the parameters “*P* = 0.05, number of threads = 10, number of random = 1000, and search for lambda.” We identified 5730 expanded gene families in the *E. breviscapus* genome, which is more than that in two other species, *C. cardunculus* (1336) and *H. annuus* (3897) in Compositae (Additional File 1: Figure S5).

In summary, we reported the genome sequencing, assembly, annotation, and evolution analysis of the *E. breviscapus*. This genome assembly will provide a valuable resource for studying the biosynthetic pathways of the medicinal components in *E. breviscapus*. This information will also help find novel bioactive compounds and improve the molecular breeding of this medicinal herb.

## Abbreviations

BUSCO: Benchmarking Universal Single-Copy Orthologs; CEGMA: Core Eukaryotic Genes Mapping Approach.

## Supplementary Material

GIGA-D-16-00144_Original_Submission.pdf

GIGA-D-16-00144_Revision_1.pdf

Response_to_reviewer_comments_Original_Submission.pdf

Reviewer_1_Report_(Original_Submission).pdf

Reviewer_2_Report_(Original_Submission).pdf

Reviewer_2_Report_(Revision_1).pdf

Supplemental material **Table S1:** Raw sequencing statistics from the Illumina platform and PacBio platform.
**Table S2:** Summary of genome assembly.
**Table S3:** Summary of transcriptomes.
**Table S4:** Statistics of repeats in the *E. breviscapus* genome.
**Table S5:** Repeat annotation of the *E. breviscapus* genome assembly.
**Table S6:** Gene annotation statistics for the *E. breviscapus* genome.
**Table S7:** Summary of non-protein-coding gene annotation in the *E. breviscapus* genome assembly.
**Figure S1:** The estimated genome size of *E. breviscapus* with flow cytometry.
**Figure S2:** Frequency distribution of the 23-mer graph.
**Figure S3:** Phylogenetic reconstruction of the *E. breviscapus* and six other plant species.
**Figure S4:** Divergence time estimation of the *E. breviscapus* and six other plant species.
**Figure S5:** Gene family expansions and contractions in the *E. breviscapus*.

## Additional File

**Table S1:** Raw sequencing statistics from the Illumina platform and PacBio platform.

**Table S2:** Summary of genome assembly.

**Table S3:** Summary of transcriptomes.

**Table S4:** Statistics of repeats in the *E. breviscapus* genome.

**Table S5:** Repeat annotation of the *E. breviscapus* genome assembly.

**Table S6:** Gene annotation statistics for the *E. breviscapus* genome.

**Table S7:** Summary of non-protein-coding gene annotation in the *E. breviscapus* genome assembly.

**Figure S1:** The estimated genome size of *E. breviscapus* with flow cytometry.

**Figure S2:** Frequency distribution of the 23-mer graph.

**Figure S3:** Phylogenetic reconstruction of the *E. breviscapus* and six other plant species.

**Figure S4:** Divergence time estimation of the *E. breviscapus* and six other plant species.

**Figure S5:** Gene family expansions and contractions in the *E. breviscapus*.

## Availability of supporting data

Sequencing reads of each sequencing library and RNA-seq data have been deposited at the National Center for Biotechnology Information with the project ID PRJNA352312. Supporting data, including alignments, annotations, and custom scripts, are available in the *GigaScience* database (GigaDB) [42]. All supplementary figures and tables are provided in Additional File 1.
