# Supplementary material for: Hybrid de novo genome assembly of the Chinese herbal fleabane Erigeron breviscapus
Source: Gigascience. 2017 Apr 18;6(6):1–7. doi: 10.1093/gigascience/gix028 (PMC5449645; doi:10.1093/gigascience/gix028)
Supplement: GIGA-D-16-00144_Original_Submission.pdf [file gix028_GIGA-D-16-00144_Original_Submission.pdf]

Manuscript
Click here to download Manuscript Erigeron breviscapusgenome.docx

Hybrid *de novo* genome assembly of the Chinese herbal fleabane *Erigeron breviscapus*

Jing Yang[1,9], Guanghui Zhang[2,9], Jing Zhang[3,9], Hui Liu[4,5], Wei Chen[1,6], Xiao Wang[4,5], Yahe Li[7], Yang Dong[1,6,8,10], Shengchao Yang[2,10]

[1]Biological Big Data College, Yunnan Agricultural University, Kunming 650201, China.

[2]National-Local Joint Engineering Research Center on Germplasm Utilization and Innovation of Chinese Medicinal Materials in Southwest China, Yunnan Agricultural University, Kunming 650201, China.

[3]NOWBIO Technology Co. Ltd, Kunming 650202, China.

[4]State Key Laboratory of Genetic Resources and Evolution, Kunming Institute of Zoology, Chinese Academy of Sciences, Kunming 650223, China.

[5]University of Chinese Academy of Sciences, Beijing 100049, China.

[6]Yunnan Research Institute for Local Plateau Agriculture and Industry, Kunming 650201, China

[7]Longjing Pharmaceutical Co. Ltd, Kunming 650228, China.

[8]College of Life Science, Kunming University of Science and Technology, Kunming, China.

[9]Co-first authors.

[10]Corresponding authors.

Abstract

Background: The plants in the *Erigeron* genus of the Compositae (Asteraceae) family are commonly called fleabanes, possibly due to the belief that certain

chemicals in these plants repel fleas. In the traditional Chinese medicine, *Erigeron breviscapus*, which is native to China, was widely used in the treatment of cerebrovascular disease. A handful of bioactive compounds, including scutellarin, 3,5-dicaffeoylquinic acid, and 3,4-dicaffeoylquinic acid, have been isolated from the plant. With the purpose of finding novel medicinal compounds and understanding their biosynthetic pathways, we propose to sequence the genome of *E. breviscapus*.

Findings: We assembled the highly heterozygous *E. breviscapus* genome using a combination of PacBio single-molecular real-time sequencing method and next-generation sequencing method on the Illumina HiSeq platform. The final draft genome is approximately 1.2 Gb, with the contig and scaffold N50 sizes of 18.8 kb and 31.5 kb, respectively. Further analyses predicted 70,214 protein-coding genes in the *E. breviscapus* genome, and 9,825 shared gene families among Compositae species.

Conclusions: The *E. breviscapus* genome provides a valuable resource for the investigation of novel bioactive compounds in this Chinese herb.

Keywords: *Erigeron breviscapus*, Illumina sequencing, PacBio sequencing.

Background

Erigeron breviscapus (also known as *dengzhanhua* in Chinese) is a perennial flower in the *Erigeron* genus of the Compositae (Asteraceae) family. Its flower head is

comprised of yellow disk florets and multiple surrounding blue to purple ray florets (Fig. 1). This species is endemic to Southwestern China, which grows in mid-altitude mountains, subalpine open slopes, grasslands and forest margins from 1000 m to 3500 m [1,2]. In the traditional Chinese medicine, *E. breviscapus* is believed to improve blood circulation and ameliorate platelet coagulation [3,4]. Since the 1980s, the herbal extracts and bioactive compounds from *E. breviscapus* have been widely used for the treatment of cerebral embolism and its complications, cerebral thrombosis, coronary heart disease, angina pectoris, acute renal failure, and nephritic syndrome [5]. At present, more than 1,000 tons of dry *E. breviscapus* are collected and used in the pharmaceutical industry each year, greatly exhausting the wild resources of this species [6,7]. In this study, we report the draft genome assembly of *E. breviscapus*. Because of the high heterozygosity of the *E. breviscapus* genome, we adopted both Illumina sequencing and PacBio single-molecular real-time sequencing in the assembly procedure.

Data description

Whole-genome shotgun sequencing of *E. breviscapus* on Illumina platform

E. breviscapus seedlings were provided by the Longjing Pharmaceutical Co. Ltd and maintained in a greenhouse at the Yunnan Agricultural University. Genomic DNA was extracted from the leaf tissues of a single *E. breviscapus* plant using the GenElute^{TM} Plant Genomic DNA Miniprep Kit (Sigma-Aldrich; St. Louis, USA). Paired-end

libraries with insert sizes ranging from 150 bp to 800 bp were constructed using NEBNext Ultra II DNA Library Prep Kit for Illumina (NEB, USA), and mate pair libraries with insert sizes from 2 kb to 20 kb were constructed using Illumina Nextera Mate Pair Library Preparation Kit (Illumina, USA). All constructed libraries were sequenced on a HiSeq 2500 platform (Illumina, USA) using either a PE-100 or PE-90 module (Additional file 1: Table S1). In total, about ~413.4 Gb raw data were generated on the Illumina platform. The raw data were initially filtered by removing reads with more than 10 % N or more than 40 bp low quality bases. Next, redundant reads resulting in duplicate base calls were filtered at a threshold of Euclidean distance ≤ 3 and mismatch rate of ≤ 0.1. Only one copy of any duplicated paired-end reads was retained. Finally, both read 1 and read 2 were removed if they contained an adapter ≥ 10 bp with a mismatch rate ≤ 0.1. This process yielded ~275.1 Gb of clean data for the *de novo* assembly of the *E. breviscapus* genome (Additional file 1: Table S1).

Single-molecule real-time sequencing of long reads on PacBio platform

Single-molecule real-time (SMRT) sequencing of long reads on a PacBio RS II platform (Pacific Biosciences, USA) was used to assist the subsequent *de novo* genome assembly process [8]. In brief, 40 μg of sheared DNA was used to construct 26 SMRT Cell libraries with an insert size of 17 kb. These libraries were sequenced in 105 SMRT DNA sequencing cells using the P6 polymerase/C4 chemistry combination,

and a data collection time of 240 min per cell. The sequencing produced about 62.4 Gb clean data, consisting of 6,802,553 reads with an average read length of 9,175 bp (Additional file 1: Table S1).

Estimation of the *E. breviscapus* genome size

The genome size of *E. breviscapus* was estimated by flow cytometry, using *Oryza sativa* Nipponbare as internal standard and propidium iodide as the stain. The result showed that the genome size of *E. breviscapus* was approximately 1.52 Gb (Additional file 1: Figure S1).

Hybrid *de novo* genome assembly of *E. breviscapus*

A hybrid genome assembly pipeline was used to overcome challenges posed by the heterozygous *E. breviscapus* genome (Fig. 2). HiSeq reads were first assembled using MaSuRCA [9] with default parameters, and also using Platanus [10] with parameters "-m 500 -k 43 -s 5 -d 0.3 -u 0.15 -c 3", resulting in two contig assemblies. The Platanus-generated contigs, together with PacBio reads, were used to generate a third contig assembly by DBG2OLC with default parameters [11]. The three different contig assemblies were merged together by Minimus2 using default parameters [12]. To eliminate possible errors of the merged contig assembly, Bowtie2 [13] was used to align HiSeq reads back to this assembly. The result was further polished by PICARD and GATK using default parameters [14,15]. Polished contigs were then used to build

scaffolds using OPERA [16] with a *k*-mer of 39. This process yielded a final draft *E. breviscapus* genome of 1.2 Gb, with a contig N50 size of 18.8 kb and a scaffold N50 size of 31.5 kb (Additional file 1: Table S2).

Evaluation of the completeness of the *E. breviscapus* genome assembly

We evaluated the completeness of the final assembly using CEGMA [17] with a set of 248 ultra-conserved core eukaryotic genes and BUSCO [18] with the Embryophyta gene set. CEGMA assessment showed that our assembly captured 240 (96.9 %) of the 248 ultra-conserved core eukaryotic genes, of which 217 (87.5 %) were complete (Table 1). BUSCO analysis showed that 80.6 % and 6.3 % of the 1440 expected embryophytic genes were identified as complete and fragmented, respectively (Table 2).

Transcriptome sequencing

Total RNA was extracted from the leaf, root, stem, and flower tissues of a cultivated *E. breviscapus* individual using Qiagen RNeasy Plant Mini Kits. Additional RNA samples of the leaf tissues were acquired from six more cultivated individuals and five wild individuals (Additional file 1: Table S3). All cultivated samples were acquired from the greenhouse and all wild samples were collected from Dali, Yunnan Province. Total RNA-seq libraries were prepared using TruSeq RNA Library Preparation Kit v2 (Illumina, CA, USA) according to the manufacturer's instructions

and subsequently sequenced on the HiSeq 2500 platform. In total, about 1.1 billion RNA-seq reads were obtained, representing ~117.6 Gb raw data. We aligned all the RNA-seq reads back to the *E. breviscapus* genome assembly using TopHat [19] with default parameters (Additional file 1: Table S3). The percentage of aligned reads ranged from 60.6 % for the root to 80.9 % for the leaf. The FPKM value was calculated for each protein-coding gene by Cufflinks using default parameters. FPKM >0.05 was used as the cutoff value to identify expressed genes.

Repeat annotation of the *E. breviscapus* genome assembly

The *E. breviscapus* genome was searched for tandem repeats using the Tandem Repeat Finder [20]. RepeatMasker and RepeatProteinMasker [21] were used against Repbase library [22] to identify known transposable element repeats. *De novo* evolved transposable element annotation was performed using RepeatModeler [21] and LTR FINDER [23]. The combined results show that the total length of repeated sequences is about 664.2 Mb, accounting for ~54.58 % of the *E. breviscapus* genome assembly (Additional file 1: Table S4 and S5).

Gene prediction

We used multiple methods to annotate protein-coding genes in the *E. breviscapus* genome, including homology-based predictions, *de novo* predictions, and transcriptome-based predictions. For homology-based predictions, protein sequences

of *Arabidopsis thaliana*, *Fragaria vesca*, *Malus domestica*, *Oryza sativa*, *Prunus persica* and *Vitis vinifera* were obtained from Phytozome v9.1 (http://www.phytozome.net/), *Pyrus communis* from Genome Database for Rosaceae (https://www.rosaceae.org), and *Prunus mume* from NCBI (ftp://ftp.ncbi.nih.gov/genomes/Prunus_mume). First, query sequences were subjected to TBLASTN analysis with a cutoff E-value of $1e^{-5}$. BLAST hits corresponding to reference proteins were concatenated by Solar [24] (The Beijing Genomics Institute (BGI) development) after low-quality records were removed. The genomic sequence of each reference protein was extended upstream and downstream by 2,000 bp to represent a protein-coding region. GeneWise [25] was used to predict gene structure contained in each protein region. For *de novo* predictions, AUGUSTUS [26], GENSCAN [27] and glimmerHMM [28] analyses were performed on the repeat-masked genome, with parameters trained from *A. thaliana*. For transcriptome-based predictions, RNA-seq data from the leaves of six cultivated individuals were used for gene annotation, processed by Tophat and Cufflinks [19]. The homology, *de novo* and transcriptomic-based predicted gene sets were merged to form a comprehensive and non-redundant reference gene set using EVidenceModeler [29]. Our analysis indicates that the *E. breviscapus* genome contains 70,214 protein-coding genes with an average CDS length of 839 bp (Additional file 1: Table S6).

Non-coding RNA annotation

tRNAscan-SE [30] with default parameters for eukaryotes was used for tRNA annotation. Homology-based rRNA annotation was performed by mapping plant rRNAs to the *E. breviscapus* genome using BLASTN with parameters of "E-value = $1e^{-5}$". miRNA and snRNA genes were predicted by INFERNAL [31] using the Rfam database (release 11.0) [32]. The final results include 504 miRNAs, 751 tRNAs, 159 rRNAs, and 385 snRNAs (Additional file 1: Table S7).

Gene family clustering analysis

To identify and estimate the number of potential orthologous gene families between *E. breviscapus*, *Helianthus annuus*, *Cynara cardunculus*, *Solanum tuberosum*, *Solanum lycopersicum*, *V. vinifera*, and *O. sativa*, we applied the OrthoMCL pipeline [33] using standard settings (BLASTP E-value < $1e^{-5}$) to compute the all-against-all similarities. Gene sequences from *S. tuberosum*, *S. lycopersicum*, *V. vinifera*, and *O. sativa* were downloaded from Phytozome v11.0. Gene sequences from *H. annuus* and *C. cardunculus* were downloaded from Sunflower Genome Database (http://www.sunflowergenome.org) and Globe artichoke GBrowse (http://gviewer.gc.ucdavis.edu/cgi-bin/gbrowse/Artichoke_v1_1), respectively. Among the total 19,565 *E. breviscapus* gene families, 5,501 (28.1%) appear to be lineage specific. There are 9,825 (50.2%) gene families shared among Compositae species including *E. breviscapus*, *H. annuus*, and *C. cardunculus*. In addition, *E.*

breviscapus shared 7,957 (40.7%) gene families with *S. tuberosum* (Fig. 3).

Phylogenetic Tree Construction and Divergence Time Estimation

All 413 single-copy orthologous genes identified in the gene family clustering analysis from the *S. lycopersicum*, *V. vinifera, O. sativa, E. breviscapus, H. annuus*, *C. cardunculus*, and *S. tuberosum* were used to construct a phylogenetic tree. Orthologous genes from the seven species were aligned using MUSCLE with default settings [32] for each gene. Four-fold degenerate sites were extracted from each gene and concatenated into a ‘‘super gene’’ for each species. PhyML [35] was used to reconstruct phylogenetic trees between species. We implemented a Monte Carlo Markov chain (MCMC) algorithm for the estimation of divergence times using the program MCMCtree from the PAML package [36]. The result showed that *E. breviscapus* shared a closer phylogenetic relationship with *H. annuus* than *C. cardunculus* in the Compositae family (Additional file 1: Figure S2). The estimated divergence time was 29.4 million years ago between *E. breviscapus* and *H. annuus* (Additional file 1: Figure S3).

Expansion and Contraction of Gene Families

CAFE [37] is a tool for analyzing the evolution of gene family size based on the stochastic birth and death model. With the calculated phylogeny and the divergence time, this software was applied to identify gene families that had undergone

expansion and/or contraction in *S. lycopersicum*, *V. vinifera*, *O. sativa*, *E. breviscapus*, *H. annuus*, *C. cardunculus*, and *S. tuberosum* with the parameters “p-value = 0.05, number of threads = 10, number of random = 1000, and search for lambda”. We identified 10,845 expanded gene families in the *E. breviscapus* genome, which is more than that in two other species *C. cardunculus* (1,059) and *H. annuus* (3,480) in Compositae (Additional file 1: Figure S4).

In summary, we reported the genome sequencing, assembly, annotation, and evolution analysis of the *E. breviscapus*. This genome assembly will provide a valuable resource for studying the biosynthetic pathways of the medicinal components in *E. breviscapus*. This information will also help find novel bioactive compounds, and improve the molecular breeding of this medicinal herb.

Availability of supporting data

Sequencing reads of each sequencing library and RNA-seq data have been deposited at NCBI with the project ID PRJNA352312. Supporting data are also available in the GigaScience database, GigaDB [38]. All supplementary figures and tables are provided in Additional file 1.

Additional file

Additional file 1: Supplemental tables and figures. Table S1. Raw sequencing

statistics from the Illumina platform and PacBio platform. **Table S2.** Summary of genome assembly. **Table S3.** Summary of transcriptomes. **Table S4.** Statistics of repeats in the *E. breviscapus* genome. **Table S5.** Repeat annotation of the *E. breviscapus* genome assembly. **Table S6.** Gene annotation statistics for the *E. breviscapus* genome. **Table S7.** Summary of non-protein-coding gene annotation in the *E. breviscapus* genome assembly. **Figure S1.** The estimated genome size of *E. breviscapus* with flow cytometry. **Figure S2.** Phylogenetic reconstruction of the *E. breviscapus* and six other plant species. **Figure S3.** Divergence time estimation of the *E. breviscapus* and six other plant species. **Figure S4.** Gene family expansions and contractions in the *E. breviscapus*.

Abbreviations

CDS: Coding DNA sequence; NCBI: National Center for Biotechnology Information; CEGMA: Core Eukaryotic Genes Mapping Approach; BUSCO: Benchmarking Universal Single-Copy Orthologs.

Funding

This work was support by the pilot project for establishing new socialized service system by agricultural science and education combination in Yunnan Province (Medical Plant Unit) (2014NG003) and National Natural Science Foundation of China (81260614).

Competing interests

The authors declare that they have no competing interests.

Authors' contributions

WC, YD, GZ and SY designed the study. HL assembled the genome. JY, JZ analyzed the data. JY, WC and YD wrote the manuscript. All authors read and approved the final manuscript.

Acknowledgements

We thank Longjing Pharmaceutical Co. Ltd for providing samples of *E. breviscapus* plant.

Author details

[1]Biological Big Data College, Yunnan Agricultural University, Kunming 650201, China. [2]National-Local Joint Engineering Research Center on Germplasm Utilization and Innovation of Chinese Medicinal Materials in Southwest China, Yunnan Agricultural University, Kunming 650201, China. [3]NOWBIO Technology Co. Ltd, Kunming 650202, China. [4]State Key Laboratory of Genetic Resources and Evolution, Kunming Institute of Zoology, Chinese Academy of Sciences, Kunming 650223, China.[5]University of Chinese Academy of Sciences, Beijing 100049, China. [6] Yunnan Research Institute for Local Plateau Agriculture and Industry, Kunming 650201, China. [7]Longjing Pharmaceutical Co. Ltd, Kunming 650228, China. [8]College of Life Science, Kunming University of Science and Technology, Kunming, China. [9]Co-first authors. [10]Corresponding authors.

References

1. Lin R, Chen Y, Shi Z. Flora Reipublicae Popularis Sinicae. Vol. 74. Science Press. Beijing;1985. p. 308–9.
2. Li X, Zhang S, Yang Z, Song K, Yi T. Conservation genetics and population diversity of *Erigeron breviscapus*, (Asteraceae), an important Chinese herb. Biochem Syst Ecol. 2013;49(2):156-66.
3. Sheng J, Zhao P, Huang Z. Influence of deng zhan xi xin (*Erigeron breviscapus*) on thrombolytic treatment during acute coronary thrombosis by affecting function of blood platelet and coagulation. Chin J Cardiol. 1999;27(2):115-7.
4. Liu H, Tang X, Wang Y, Tang R, Yang X, Fu X, et al. Effects of scutellarin on rat cerebral blood flow determined by laser speckle imagine system. Chin Hosp Pharm J. 2010;30(9):719-722.
5. Sun H. A Drug for Treating Cardio-Cerebrovascular Diseases-Phenolic Compounds of *Erigeron breviscapus*. PROG CHEM. 2009;21(1):77-83.
6. Yu H, Chen Z. Study on artificial culture of *Erigeron breviscapus*. Acta Bot Yunnanica. 2002;24,115–20.
7. Li X, Song K, Yang J, Yi T. Isolation and Characterization of 11 New Microsatellite Loci in *Erigeron breviscapus* (Asteraceae), an Important Chinese Traditional Herb. Int J Mol Sci. 2011;12(10):7265-70.
8. Eid J, Fehr A, Gray J, Luong K, Lyle J, Otto G, et al. Real-time DNA sequencing from single polymerase molecules. Science. 2009;323:133–8.

9. Zimin AV, Marçais G, Puiu D, Roberts M, Salzberg SL, Yorke JA. The MaSuRCA genome assembler. Bioinformatics. 2013;29(21):2669-77.

10. Kajitani R, Toshimoto K, Noguchi H, Toyoda A, Ogura Y, Okuno M, et al. Efficient de novo assembly of highly heterozygous genomes from whole-genome shotgun short reads. Genome Res. 2014;24:1384–95.

11. Ye C, Hill C, Ruan J. DBG2OLC: Efficient assembly of large genomes using the compressed overlap graph. arXiv preprint arXiv:1410.2801, 2014.

12. Treangen TJ, Sommer DD, Angly FE, Koren S, Pop M. Next generation sequence assembly with AMOS. Curr Protoc Bioinformatics. 2011;CHAPTER: Unit11.8–Unit11.8.

13. Langmead B, Salzberg S. Fast gapped-read alignment with Bowtie 2. Nature Methods. 2012;9:357-359.

14. McKenna A, Hanna M, Banks E, Sivachenko A, Cibulskis K, Kernytsky A, et al. The Genome Analysis Toolkit: a MapReduce framework for analyzing next-generation DNA sequencing data. Genome Res. 2010;20(9): 1297-1303.

15. DePristo MA, Banks E, Poplin R, Garimella KV., Maguire JR., Hartl C, et al. A framework for variation discovery and genotyping using next-generation DNA sequencing data. Nat Genet. 2011;43(5): 491-8.

16. Gao S, Nagarajan N, Sung WK. Opera: Reconstructing Optimal Genomic Scaffolds with High-Throughput Paired-End Sequences. J Comput Biol. 2011;18(11):1681-91.

17. Parra G, Bradnam K, Korf I. CEGMA: a pipeline to accurately annotate core genes in eukaryotic genomes. Bioinformatics. 2007;23:1061–7.

18. Simão FA, Waterhouse RM, Ioannidis P, Kriventseva EV, Zdobnov EM. BUSCO: assessing genome assembly and annotation completeness with single-copy orthologs. Bioinformatics. 2015;31(19):3210-2.

19. Trapnell C, Roberts A, Goff L, Pertea G, Kim D, Kelley DR, Pimentel, et al. Differential gene and transcript expression analysis of RNA-seq experiments with TopHat and Cufflinks. Nat Prot. 2012;7:562-78.

20. Benson, G. Tandem repeats finder: a program to analyze DNA sequences. Nucleic Acids Res. 1999;27:573–80.

21. Tarailo-Graovac M, Chen N. Using RepeatMasker to identify repetitive elements in genomic sequences. Curr Protoc Bioinformatics. 2009;3:4–14.

22. Jurka J, Kapitonov VV, Pavlicek A, Klonowski P, Kohany O, Walichiewicz J, et al. Repbase Update, a database of eukaryotic repetitive elements. Cytogenet. Genome Res. 2005;110(1-4):462-7.

23. Xu Z, Wang H. LTR_FINDER: an efficient tool for the prediction of full-length LTR retrotransposons. Nucleic Acids Res. 2007;35:W265–8.

24. Li X, Kui L, Zhang J, Xie Y, Wang L, Yan Y, et al. Improved hybrid *de novo* genome assembly of domesticated apple (*malus x domestica*). GigaScience, 2016;5:35.

25. Birney E, Durbin R. Using GeneWise in the Drosophila annotation experiment.

Genome Res. 2000;10:547–8.

26. Stanke M, Keller O, Gunduz I, Hayes A, Waack S, Morgenstern, B. AUGUSTUS: ab initio prediction of alternative transcripts. Nucleic Acids Res. 2006;34(suppl 2):W435-W439.

27. Cai Y, Gonzalez JV, Liu Z, Huang T. Computational systems biology methods in molecular biology, chemistry biology, molecular biomedicine, and biopharmacy. Biomed Res Int. 2014;2014:746814.

28. Majoros WH, Pertea M, Salzberg SL. TigrScan and GlimmerHMM: two open source ab initio eukaryotic gene-finders. Bioinformatics. 2004;20(16):2878-9.

29. Haas BJ, Salzberg SL, Zhu W, Pertea M, Allen JE, Orvis J, et al. Automated eukaryotic gene structure annotation using EVidenceModeler and the Program to Assemble Spliced Alignments. Genome Biol. 2008,9(1):1.

30. Lowe TM, Eddy SR. tRNAscan-SE: a program for improved detection of transfer RNA genes in genomic sequence. Nucleic Acids Res. 1997;25:955–64.

31. Nawrocki EP, Kolbe DL, Eddy SR. Infernal 1.0: inference of RNA alignments. Bioinformatics. 2009;25:1335–7.

32. Gardner PP, Daub J, Tate J, Moore BL, Osuch IH, Griffiths-Jones S, et al. Rfam: Wikipedia, clans and the ‘‘decimal’’ release. Nucleic Acids Res. 2011;39 suppl 1:D141-D145.

33. Li L, Stoeckert CJ, Roos DS. OrthoMCL: Identification of Ortholog Groups for Eukaryotic Genomes. Genome Res. 2003;13:2178–89.

34. Edgar RC. MUSCLE: multiple sequence alignment with high accuracy and high throughput. Nucleic Acids Res. 2004;32(5):1792-7.

35. Guindon S, Dufayard JF, Lefort V, Anisimova M, Hordijk W, Gascuel O. New algorithms and methods to estimate maximum-likelihood phylogenies: assessing the performance of PhyML 3.0. Systematic Biology, 2010;59(3):307-21.

36. Yang Z. PAML 4: phylogenetic analysis by maximum likelihood. Mol Biol Evol. 2007;24(8):1586-91.

37. De Bie T, Cristianini N, Demuth JP, Hahn MW. CAFE: a computational tool for the study of gene family evolution. Bioinformatics. 2006;22(10):1269-71.

38. Yang J, Zhang G, Zhang J, Liu H, Chen W, Wang X, et al. Supporting data for "Hybrid *de novo* genome assembly of the Chinese herba fleabane *Erigeron breviscapus*". GigaScience Database 2016.

Table 1 Statistics of the completeness of the hybrid *de novo* assembly genome of *E. breviscapus* by CEGMA.

**Group**	**Protein Num.**[a]	**Completeness (%)**[b]	**Total Num.**[c]	**Average Num.**[d]	**Ortholog (%)**[e]
Complete	217	87.50	633	2.92	82.95
Group1	58	87.88	158	2.72	77.59
Group2	49	87.50	126	2.57	77.55
Group3	53	86.89	171	3.23	96.23
Group4	57	87.69	178	3.12	80.70
Partial	240	96.77	856	3.57	89.58
Group1	63	95.45	206	3.27	85.71
Group2	55	98.21	185	3.36	83.64
Group3	59	96.72	232	3.93	98.31
Group4	63	96.92	233	3.70	90.48

[a] Protein Num.: Number of 248 ultra-conserved core eukaryotic genes (CEGs) present in the *E. breviscapus* genome.

[b] Completeness (%) : Percentage of 248 ultra-conserved CEGs present in the *E. breviscapus* genome.

[c] Total Num. : Total number of CEGs including putative orthologs present in the *E. breviscapus* genome.

[d] Average Num : Average number of orthologs per CEG.

[e] Ortholog (%) : Percentage of detected CEGs that have more than one ortholog.

Table 2 Statistics of the completeness of the hybrid *de novo* assembly genome of *E. breviscapus* by BUSCO.

BUSCO benchmark	Number	Percentage (%)
Total BUSCO groups searched	1440	-
Complete BUSCOs	1161	80.63
Complete and single-copy BUSCOs	635	44.10
Complete and duplicated BUSCOs	526	36.53
Fragmented BUSCOs	90	6.25
Missing BUSCOs	189	13.13

Figure Legend

Fig. 1 Example of the *E. breviscapus* (image from Shengchao Yang).

Fig. 2 Assembly pipeline for the *E. breviscapus* genome.

Fig. 3 Venn diagram showing unique and shared gene families among four sequenced dicotyledonous species.

Figure 1

Click here to download Figure
Figure 1.pdf

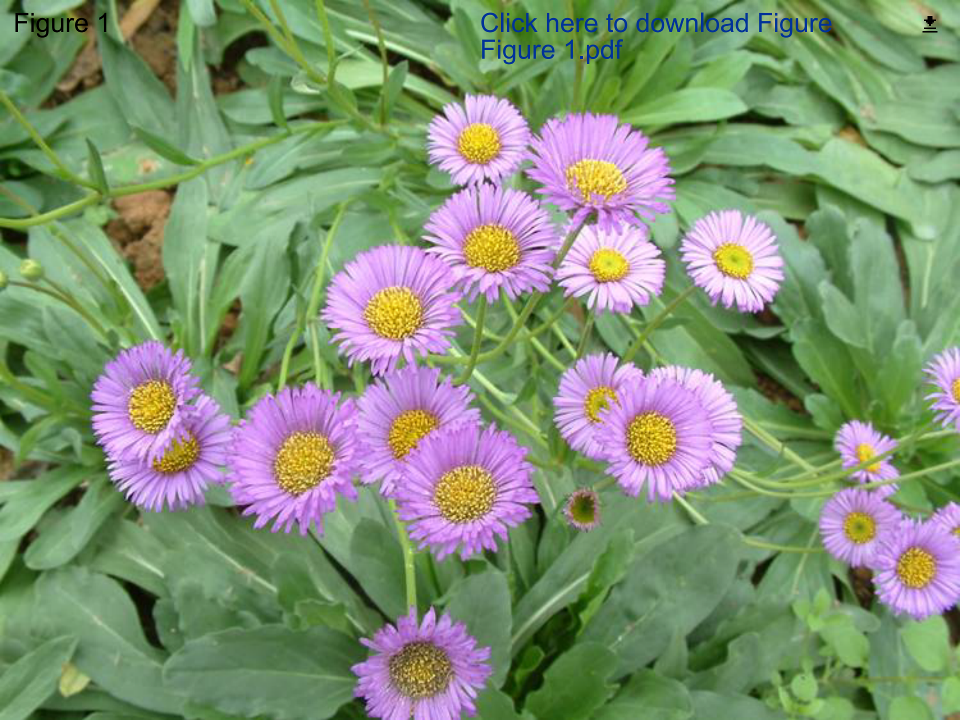

Figure 2

Click here to download Figure Figure 2.pdf

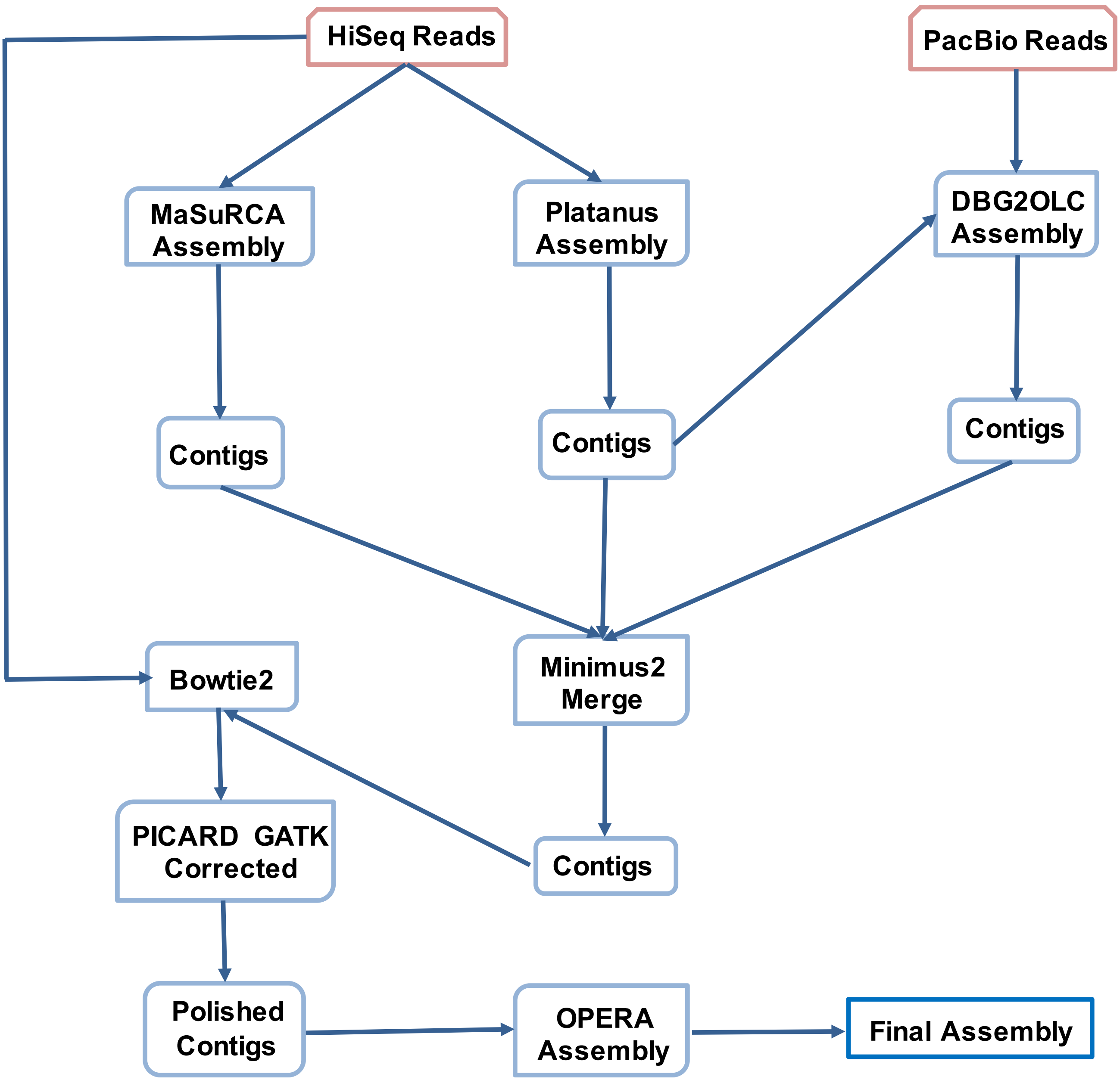

Figure 3

Click here to download Figure Figure 3.pdf

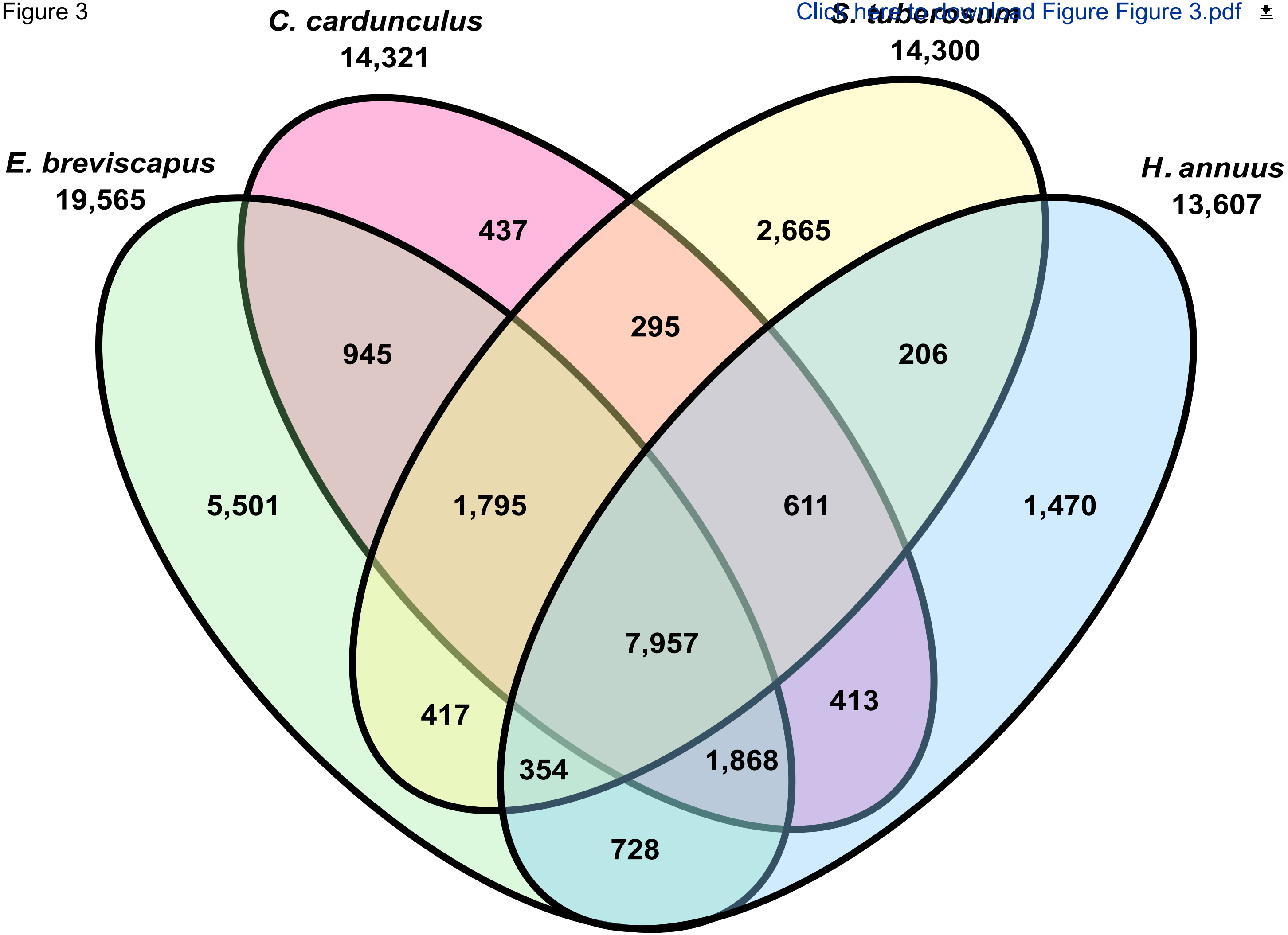

Supplementary Figure 1

Click here to access/download
Supplementary Material
Supplementary Figure 1.pdf

Supplementary Figure 2

Click here to access/download
Supplementary Material
Supplementary Figure 2.pdf

Supplementary Figure 3

Click here to access/download
Supplementary Material
Supplementary Figure 3.pdf

Supplementary Figure 4

Click here to access/download
Supplementary Material
Supplementary Figure 4.pdf

Supplementary Material table

Click here to access/download
Supplementary Material
Supplementary Material.docx
