# Supplementary material for: Hybrid de novo genome assembly of the Chinese herbal fleabane Erigeron breviscapus
Source: Gigascience. 2017 Apr 18;6(6):1–7. doi: 10.1093/gigascience/gix028 (PMC5449645; doi:10.1093/gigascience/gix028)
Supplement: Supplemental material — Table S1: Raw sequencing statistics from the Illumina platform and PacBio platform. Table S2: Summary of genome assembly. Table S3: Summary of transcriptomes. Table S4: Statistics of repeats in the E. breviscapus genome. Table S5: Repeat annotation of the E. breviscapus genome assembly. Table S6: Gene annotation statistics for the E. breviscapus genome. Table S7: Summary of non-protein-coding gene annotation in the E. breviscapus genome assembly. Figure S1: The estimated genome size of E. breviscapus with flow cytometry. Figure S2: Frequency distribution of the 23-mer graph. Figure S3: Phylogenetic reconstruction of the E. breviscapus and six other plant species. Figure S4: Divergence time estimation of the E. breviscapus and six other plant species. Figure S5: Gene family expansions and contractions in the E. breviscapus. [file gix028_Supp.zip › Supplementary Material.pdf]

Additional tables

Table S1. Raw sequencing statistics from the Illumina platform and PacBio platform

Library type	Instrument	Insert Size (bp)	Library ID	Reads Length (bp)	Raw reads (Gb)	Clean reads (Gb)
Illumina paired-end	HiSeq 2500	150	DZH-150_NoIndex_L006	100_100	38.68	32.66
		300	DZH-300_NoIndex_L001	100_100	27.67	20.41
		300	DZH-300_NoIndex_L002	100_100	27.80	20.51
		480	DZH480_NoIndex_L005	100_100	46.87	41.14
		480	DZH-480_NoIndex_L007	100_100	36.76	32.61
		480	DZH480_NoIndex_L007	100_100	45.70	37.72
		800	DZH800_NoIndex_L006	100_100	50.33	33.27
		800	DZH-800_NoIndex_L003	100_100	46.69	30.35
Illumina mate pair	HiSeq 2500	2,000	RDZHheqDMDDWAAPEI-89	90_90	33.34	10.38
		5,000	RDZHheqDMDDLAAPEI-13	90_90	21.45	4.16
		10,000	DZHheqDNFDTAAPEI-101	90_90	19.22	6.47
		20,000	DZHheqDNMDUAAPEI-23	90_90	18.87	5.38
SMRT cell	PacBio RS II	17,000	DZH_Subreads	9,175	67.35	62.42
Total	-	-	-	-	480.73	337.48

Table S2. Summary of the final genome assembly

V1.0 assembly	Scaffold	Contig
Sequence Number	462,622	464,088
Total Length (bp)	1,217,085,526	1,019,229,613
Max. Length (bp)	341,393	279,114
Min. Length (bp)	100	100
N50 (bp)	31,461	18,821
N90 (bp)	793	652

Table S3. Summary of transcriptomes

Tissue	Type	Library ID	Raw reads Number	Raw data (Gb)	% of aligned reads	Reads mapped outside of the annotated regions
Root	Cultivated	gen_L008	54,509,720	8.18	60.6%	7,605,225
Stem	Cultivated	jing_L008	66,398,062	9.96	64.4%	9,232,370
Flower	Cultivated	hua_L008	71,525,834	10.73	62.7%	9,944,887
Leaf	Cultivated	ye_L008	54,417,070	8.16	63.2%	7,412,086
		Q2	74,689,546	6.72	78.0%	11,956,304
		Q6	72,025,960	6.48	79.2%	11,565,331
		Q8	60,520,480	5.45	79.5%	10,087,373
		Q19	77,433,078	6.97	80.9%	12,704,603
		Q22	78,852,740	7.10	80.1%	12,665,972
		Q29	79,090,312	7.12	80.4%	12,829,357
	Wild	W7	103,253,706	9.29	77.7%	16,084,136
		W9	87,349,656	7.86	78.5%	14,366,775
		W10	105,440,094	9.49	79.7%	16,379,452
		W17	82,214,746	7.40	80.0%	13,058,352
		W19	74,388,640	6.69	79.6%	11,993,899
Total	-	-	1,142,109,644	117.6	-	177,886,122

Table S4. Statistics of repeats in the *E. breviscapus* genome

Type	Repeat Size (bp)	% of genome
Trf	30,583,966	2.51
Repeatmasker	64,457,047	5.30
Proteinmask	42,729,889	3.51
De novo	635,614,189	52.22
Total	664,233,126	54.58

Table S5. Repeat annotation of the *E. breviscapus* genome assembly

	**Repbase TEs**		**TE proteins**		***De novo***		**Combined TEs**	
	Length (bp)	% in genome	Length (bp)	% in genome	Length (bp)	% in genome	Length (bp)	% in genome
DNA	4,682,453	0.38	6,330,054	0.52	97,212,461	7.99	103,893,665	8.54
LINE	4,617,352	0.38	11,381,388	0.94	45,268,811	3.72	56,039,327	4.60
SINE	41,179	0.00	0.00	0.00	9,328,963	0.77	9,362,099	0.77
LTR	55,311,842	4.54	36,626,018	3.01	441,323,942	36.26	460,352,234	37.82
Other	2,824	0.00	0.00	0.00	0.00	0.00	2,824	0.00
Unknown	0.00	0.00	147	0.00	97,492,165	8.01	97,492,312	8.01
Total	64,457,047	5.30	42,729,889	3.51	623,126,037	51.20	640,845,847	52.65

DNA, DNA transposons; LINE, long interspersed nuclear elements; TEs, transposable elements; SINE, short interspersed nuclear elements; LTR, long terminal repeat.

Table S6. Gene annotation statistics for the *E. breviscapus* genome

Methods		Gene Number	Avg. mRNA Length	Total Exon Number	Avg. Exon Length	Avg. CDS Length	Avg. Exon Number	Total Intron Number
**Homolog**	*A. thaliana*	41,285	2,340.96	193,792	210.10	986.19	4.69	55,931,789
	F. vesca	41,829	2,359.25	189,413	218.51	989.45	4.53	57,297,274
	M. domestica	48,392	2,085.48	199,935	215.76	891.42	4.13	57,783,369
	O. sativa	47,057	1,818.10	178,388	206.76	783.79	3.79	48,671,577
	P. mume	43,996	2,329.81	200,363	214.86	978.49	4.55	59,452,570
	P. persica	43,361	2,381.38	203,907	214.06	1,006.61	4.70	59,611,694
	P. communis	45,721	2,314.92	202,498	216.03	956.82	4.43	62,093,821
	V. vinifera	45,386	2,311.94	207,541	211.55	967.36	4.57	61,025,376
De novo	AUGUSTUS	50,307	2,239.26	232,436	215.28	994.65	4.62	62,612,769
	GENSCAN	52,888	5,646.83	234,313	194.54	861.90	4.43	253,065,767
	GlimmerHMM	62,639	1,921.03	243,298	206.48	802.01	3.88	70,094,069
	SNAP	106,905	1,001.11	306,072	197.53	565.54	2.86	46,564,885
**RNA-seq**	Q2	35,038	1,447.22	105,643	275.97	832.07	3.02	22,638,649
	Q6	37,203	1,455.77	113,905	276.36	846.14	3.06	24,009,148
	Q8	30,313	1,330.73	81,079	284.66	761.38	2.67	18,024,916
	Q19	36,097	1,429.21	110,294	270.95	827.89	3.06	22,989,333
	Q22	37,414	1,441.38	115,500	275.30	849.88	3.09	23,498,346
	Q29	36,630	1,458.59	113,236	279.88	865.21	3.09	23,026,402
**EVidenceModeler**		**37,504**	**2639.42**	**198,151**	**195.75**	**1034.26**	**5.28**	**60,199,886**

Table S7. Summary of non-protein-coding gene annotation in the *E. breviscapus* genome assembly.

Type	Copy Number	Avg. Length (bp)	Total Length (bp)	Pct. In Genome (%)
**miRNA**	**504**	**113.79**	**57,352**	**0.004712**
**tRNA**	**751**	**74.42**	**55,889**	**0.004592**
**rRNA**	**159**	**217.62**	**34,602**	**0.002843**
18s	69	376.74	25,995	0.002136
28s	64	87.81	5,620	0.000462
5.8s	26	114.88	2,987	0.000245
5s	0.00	0.00	0.00	0.000000
**snRNA**	**385**	**126.10**	**48,550**	**0.003989**
CD-box	184	109.28	20,108	0.001652
HACA-box	14	124.86	1,748	0.000144
splicing	187	142.75	26,694	0.002193
**Total**	1,799	109.17	196,393	0.016136
