# Supplementary figures and images for: Hybrid de novo genome assembly of the Chinese herbal fleabane Erigeron breviscapus
Source: Gigascience. 2017 Apr 18;6(6):1–7. doi: 10.1093/gigascience/gix028 (PMC5449645; doi:10.1093/gigascience/gix028)

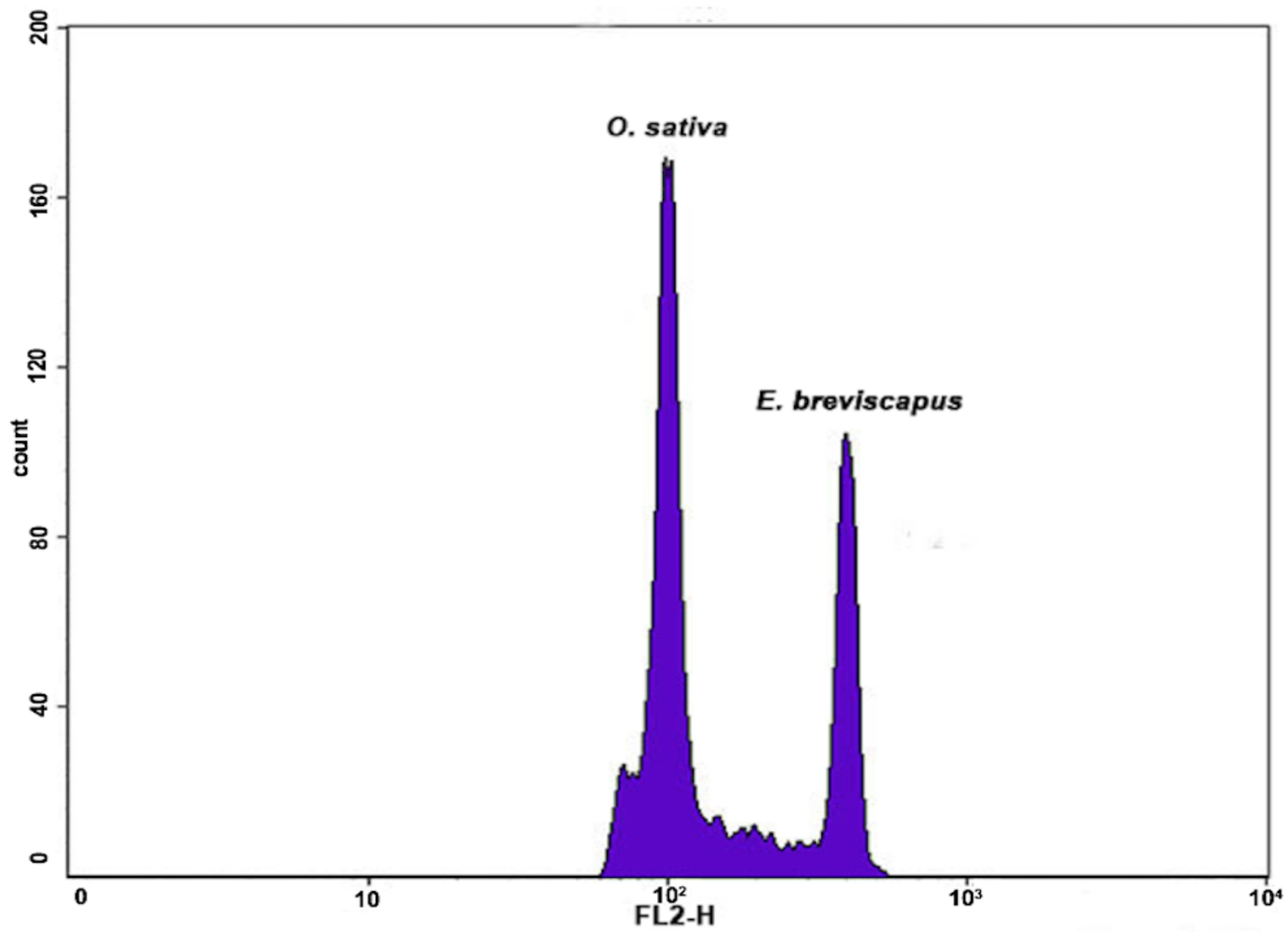

Supplement: Supplemental material — Table S1: Raw sequencing statistics from the Illumina platform and PacBio platform. Table S2: Summary of genome assembly. Table S3: Summary of transcriptomes. Table S4: Statistics of repeats in the E. breviscapus genome. Table S5: Repeat annotation of the E. breviscapus genome assembly. Table S6: Gene annotation statistics for the E. breviscapus genome. Table S7: Summary of non-protein-coding gene annotation in the E. breviscapus genome assembly. Figure S1: The estimated genome size of E. breviscapus with flow cytometry. Figure S2: Frequency distribution of the 23-mer graph. Figure S3: Phylogenetic reconstruction of the E. breviscapus and six other plant species. Figure S4: Divergence time estimation of the E. breviscapus and six other plant species. Figure S5: Gene family expansions and contractions in the E. breviscapus. [file gix028_Supp.zip › Supplementary Figure 1.pdf]

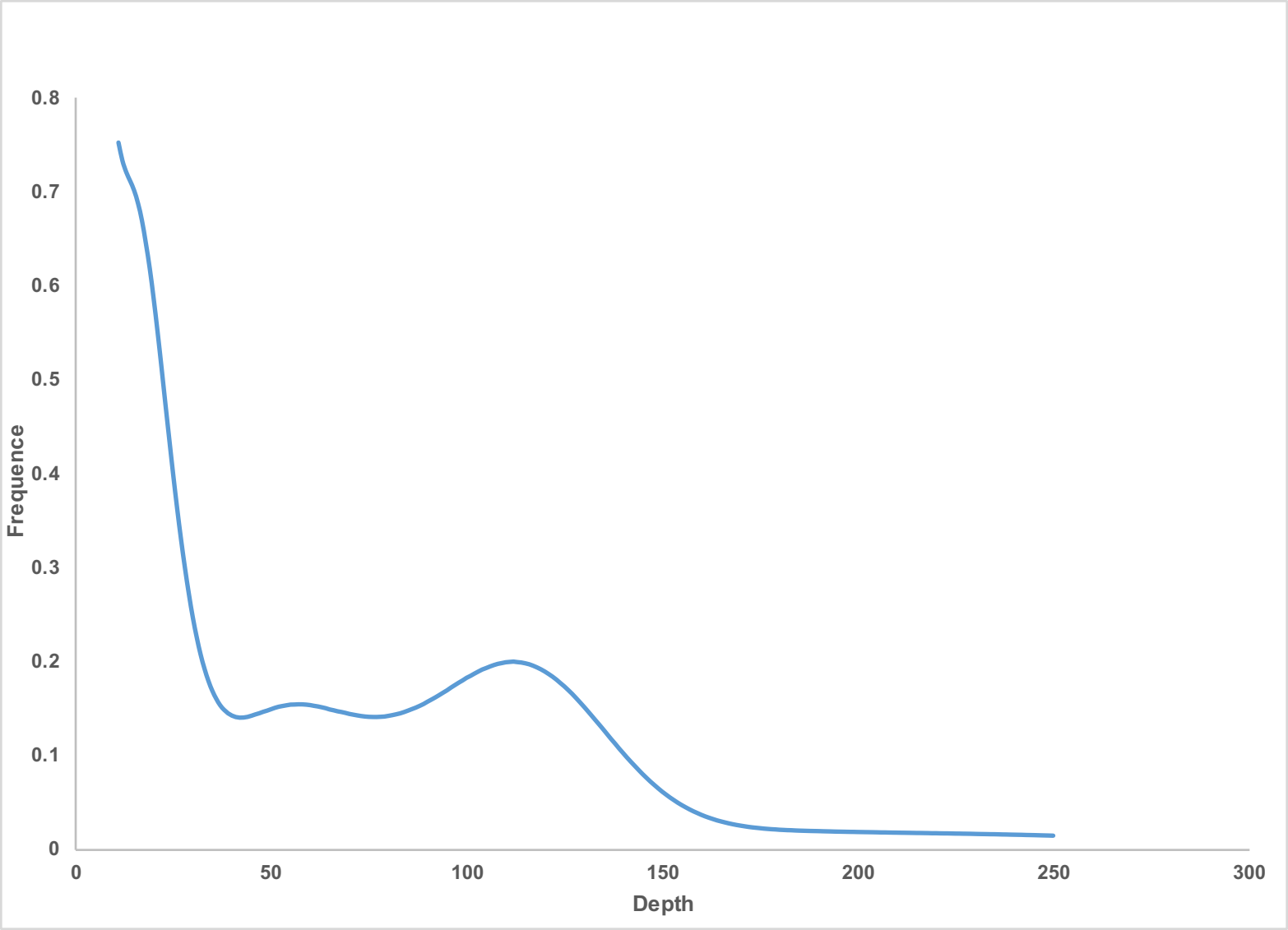

Supplement: Supplemental material — Table S1: Raw sequencing statistics from the Illumina platform and PacBio platform. Table S2: Summary of genome assembly. Table S3: Summary of transcriptomes. Table S4: Statistics of repeats in the E. breviscapus genome. Table S5: Repeat annotation of the E. breviscapus genome assembly. Table S6: Gene annotation statistics for the E. breviscapus genome. Table S7: Summary of non-protein-coding gene annotation in the E. breviscapus genome assembly. Figure S1: The estimated genome size of E. breviscapus with flow cytometry. Figure S2: Frequency distribution of the 23-mer graph. Figure S3: Phylogenetic reconstruction of the E. breviscapus and six other plant species. Figure S4: Divergence time estimation of the E. breviscapus and six other plant species. Figure S5: Gene family expansions and contractions in the E. breviscapus. [file gix028_Supp.zip › Supplementary Figure 2.pdf]

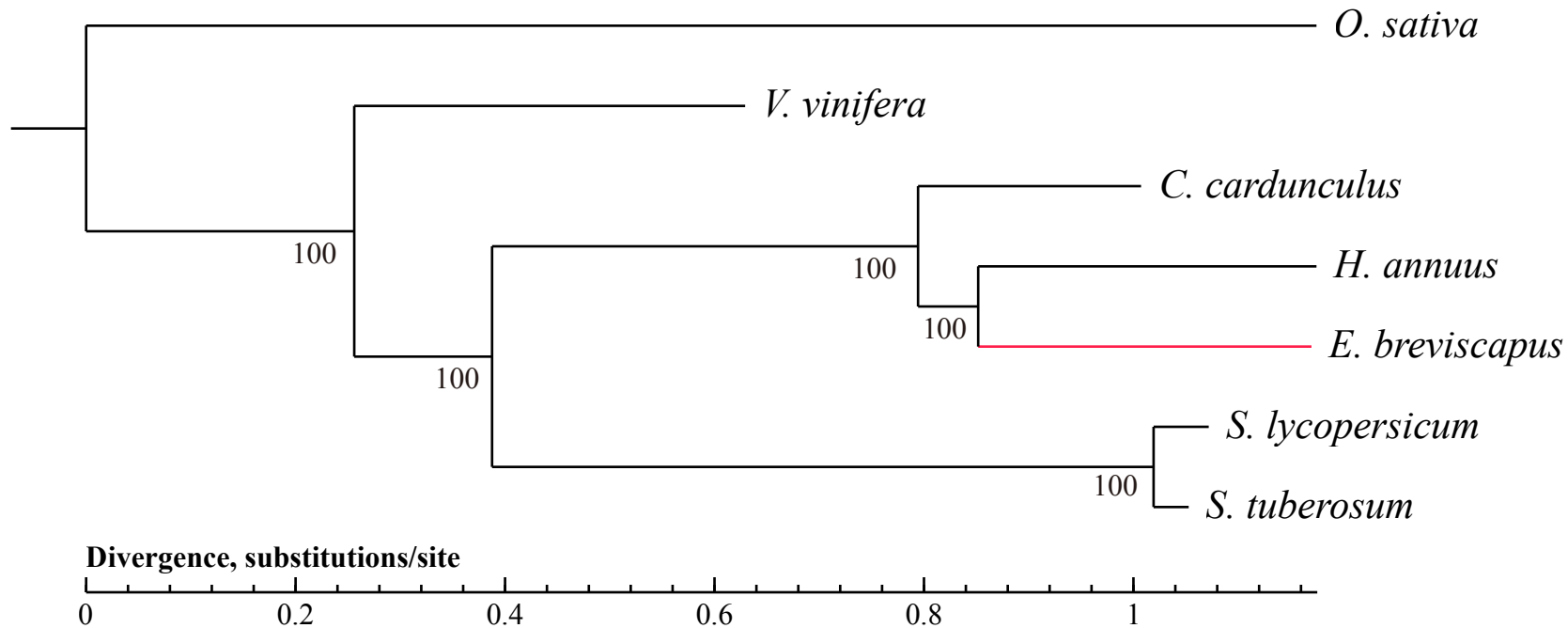

Supplement: Supplemental material — Table S1: Raw sequencing statistics from the Illumina platform and PacBio platform. Table S2: Summary of genome assembly. Table S3: Summary of transcriptomes. Table S4: Statistics of repeats in the E. breviscapus genome. Table S5: Repeat annotation of the E. breviscapus genome assembly. Table S6: Gene annotation statistics for the E. breviscapus genome. Table S7: Summary of non-protein-coding gene annotation in the E. breviscapus genome assembly. Figure S1: The estimated genome size of E. breviscapus with flow cytometry. Figure S2: Frequency distribution of the 23-mer graph. Figure S3: Phylogenetic reconstruction of the E. breviscapus and six other plant species. Figure S4: Divergence time estimation of the E. breviscapus and six other plant species. Figure S5: Gene family expansions and contractions in the E. breviscapus. [file gix028_Supp.zip › Supplementary Figure 3.pdf]

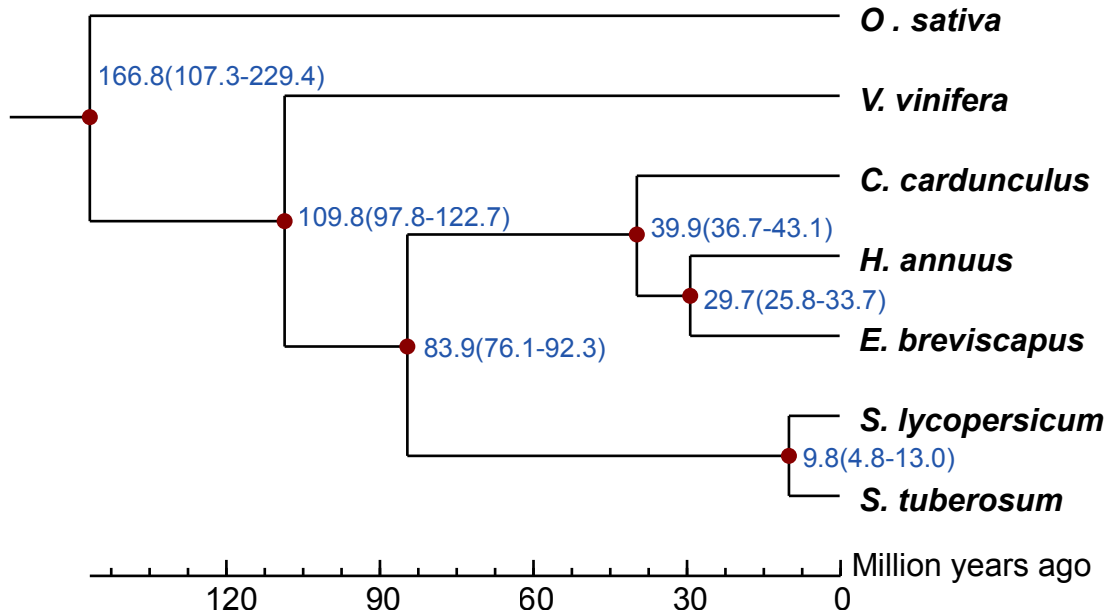

Supplement: Supplemental material — Table S1: Raw sequencing statistics from the Illumina platform and PacBio platform. Table S2: Summary of genome assembly. Table S3: Summary of transcriptomes. Table S4: Statistics of repeats in the E. breviscapus genome. Table S5: Repeat annotation of the E. breviscapus genome assembly. Table S6: Gene annotation statistics for the E. breviscapus genome. Table S7: Summary of non-protein-coding gene annotation in the E. breviscapus genome assembly. Figure S1: The estimated genome size of E. breviscapus with flow cytometry. Figure S2: Frequency distribution of the 23-mer graph. Figure S3: Phylogenetic reconstruction of the E. breviscapus and six other plant species. Figure S4: Divergence time estimation of the E. breviscapus and six other plant species. Figure S5: Gene family expansions and contractions in the E. breviscapus. [file gix028_Supp.zip › Supplementary Figure 4.pdf]

# Gene families

Expansion / Contraction

MRCA  
(26,310)

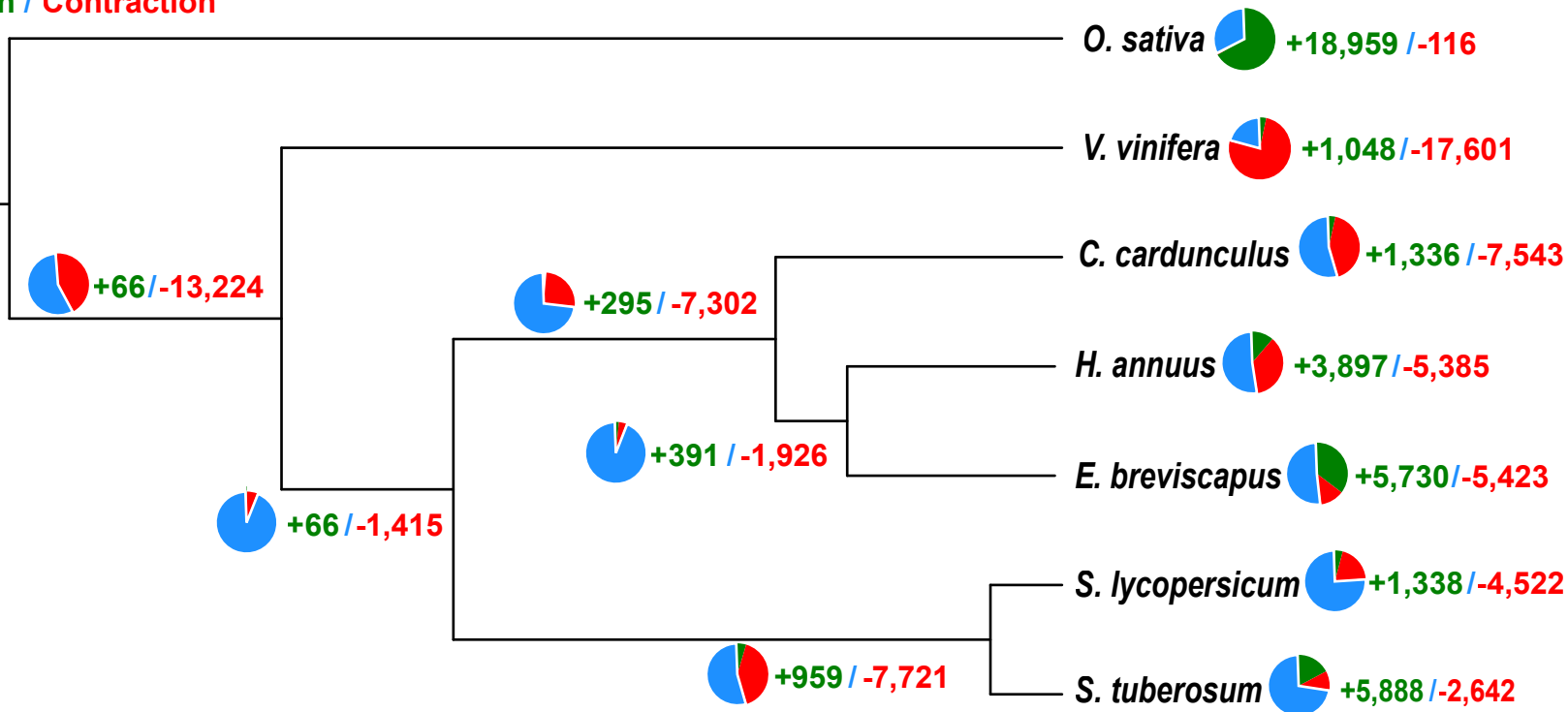

Supplement: Supplemental material — Table S1: Raw sequencing statistics from the Illumina platform and PacBio platform. Table S2: Summary of genome assembly. Table S3: Summary of transcriptomes. Table S4: Statistics of repeats in the E. breviscapus genome. Table S5: Repeat annotation of the E. breviscapus genome assembly. Table S6: Gene annotation statistics for the E. breviscapus genome. Table S7: Summary of non-protein-coding gene annotation in the E. breviscapus genome assembly. Figure S1: The estimated genome size of E. breviscapus with flow cytometry. Figure S2: Frequency distribution of the 23-mer graph. Figure S3: Phylogenetic reconstruction of the E. breviscapus and six other plant species. Figure S4: Divergence time estimation of the E. breviscapus and six other plant species. Figure S5: Gene family expansions and contractions in the E. breviscapus. [file gix028_Supp.zip › Supplementary Figure 5.pdf]
