# Supplementary material for: Hybrid de novo genome assembly of the Chinese herbal fleabane Erigeron breviscapus
Source: Gigascience. 2017 Apr 18;6(6):1–7. doi: 10.1093/gigascience/gix028 (PMC5449645; doi:10.1093/gigascience/gix028)
Supplement: Response_to_reviewer_comments_Original_Submission.pdf [file gix028_Response_to_reviewer_comments_Original_Submission.pdf]

Reviewer #1
Point 1: There is only one question or point that I would like to suggest a sentence or two in the manuscript: How many RNA-Seq reads are mapped outside of the annotated regions?
Reply: According to our calculation with HTSeq, about 177,886,122 RNA-Seq reads in total are mapped outside of the annotated region. The exact number for each individual sample is provided in Additional file 1: Table S3. We added relevant information to our manuscript in lines 149-151.

Reviewer #2
Point 1: Please provide an estimated SNP rate and/or a k-mer frequency figure to back up the claim that the genome is highly heterozygous.
Reply: We performed k-mer analysis according to the suggestion, and the result confirmed the heterozygosity of the E. breviscapus genome. The k-mer frequency figure is shown in Additional file 1: Figure S2. Please see lines 99-104 for relevant information.

Point 2: In line 106, the authors hint that merging the three assemblies with Minimus2 could lead to mis-assemblies and other errors, but they do not describe in enough detail how merging errors were prevented or detected and corrected using Bowtie alignments.
Reply: We elaborated the procedure in lines 116-123 in the revised manuscript.

Point 3: More detail is also needed about the "further polishing" of the merged assembly using Picard and GATK. The actual programs used from the Picard and GATK packages also need to be stated.
Reply: We elaborated the procedure in lines 116-123 in the revised manuscript.

Point 4: 70,214 protein coding genes seems higher than expected. From Table S6, the individual inputs into EVM suggest 35-45k. This suggests to me that some filtering is needed or there is an issue with the EVM run itself.
Reply: We appreciate the reviewer for spotting this issue. We rechecked our protein-coding gene annotation results and identified a missing account of SNAP result in Table S6. This negligence probably was introduced during the preparation of the corresponding table. The SNAP result is now added back to Table S6. Because the genome of the E. breviscapus is highly heterozygous, we agree with the reviewer that a more stringent filtering process is needed. To address this issue, we filtered out: (1) the genes containing one exon, which were not supported by the transcriptome. (2) small genes with a protein-coding region <150 bp. (3) genes with incomplete ORFs. (4) genes with stop codons in the middle of the ORFs. This process yielded 37,504 protein-coding genes, down from the initial prediction (70,214). Please see lines 187-192.

Minor Comment 1: Lines 73-79: The tools or scripts used for the read filtering steps should be stated.
Reply: We stated the scripts at the corresponding places.

Minor Comment 2: In general, the versions of the software used and the parameters should be stated.
Reply: Please see our revisions throughout the manuscript.

Minor Comment 3: The OrthoMCL and CAFE output file should be included in supporting data deposited to GigaDB.
Minor Comment 4: Since FPKMs were calculated for the annotation from the RNA-Seq alignments (line 134), the FPKM matrix should also be deposited the GigaDB.
Reply: We uploaded the OrthoMCL, CAFE output files and the FPKM matrix to the GigaDB.
