# Supplementary material for: Hybrid de novo genome assembly of the Chinese herbal fleabane Erigeron breviscapus
Source: Gigascience. 2017 Apr 18;6(6):1–7. doi: 10.1093/gigascience/gix028 (PMC5449645; doi:10.1093/gigascience/gix028)
Supplement: Reviewer_2_Report_(Original_Submission).pdf [file gix028_Reviewer_2_Report_(Original_Submission).pdf]

Reviewer Report

Title: "Hybrid de novo genome assembly of the Chinese herbal fleabane Erigeron breviscapus"

Version: Original Submission **Date:** 1/22/2017

Reviewer name: John Hamilton

Reviewer Comments to Author:

The manuscript describes the sequencing and assembly of the medicinal plant Erigeron creviscapus. Also described for E. creviscapus and six other plant species, are the identification of orthologous groups, creation of a phylogenetic tree, estimation of the divergence time, and a gene family size analysis.

Major comments:

1. Please provide an estimated SNP rate and/or a k-mer frequency figure to back up the claim that the genome is highly heterozygous.

2. In line 106, the authors hint that merging the three assemblies with Minimus2 could lead to mis-assemblies and other errors, but they do not describe in enough detail how merging errors were prevented or detected and corrected using Bowtie alignments.

3. More detail is also needed about the "further polishing" of the merged assembly using Picard and GATK. The actual programs used from the Picard and GATK packages also need to be stated.

4. 70,214 protein coding genes seems higher than expected. From Table S6, the individual inputs into EVM suggest 35-45k. This suggests to me that some filtering is needed or there is an issue with the EVM run itself.

Minor Comments:

1. Lines 73-79: The tools or scripts used for the read filtering steps should be stated.

2. In general, the versions of the software used and the parameters should be stated.

3. The OrthoMCL and CAFE output file should be included in supporting data deposited to GigaDB.

4. Since FPKMs were calculated for the annotation from the RNA-Seq alignments (line 134), the FPKM matrix should also be deposited the GigaDB.

Level of Interest

Please indicate how interesting you found the manuscript: An article of limited interest

Quality of Written English

Please indicate the quality of language in the manuscript: Acceptable

Declaration of Competing Interests

Please complete a declaration of competing interests, considering the following questions:

- Have you in the past five years received reimbursements, fees, funding, or salary from an organisation that may in any way gain or lose financially from the publication of this manuscript, either now or in the future?
- Do you hold any stocks or shares in an organisation that may in any way gain or lose financially from the publication of this manuscript, either now or in the future?
- Do you hold or are you currently applying for any patents relating to the content of the manuscript?
- Have you received reimbursements, fees, funding, or salary from an organization that holds or has applied for patents relating to the content of the manuscript?
- Do you have any other financial competing interests?
- Do you have any non-financial competing interests in relation to this paper?

If you can answer no to all of the above, write 'I declare that I have no competing interests' below. If your reply is yes to any, please give details below.

I declare that I have no competing interests

I agree to the open peer review policy of the journal. I understand that my name will be included on my report to the authors and, if the manuscript is accepted for publication, my named report including any attachments I upload will be posted on the website along with the authors' responses. I agree for my report to be made available under an Open Access Creative Commons CC-BY license (http://creativecommons.org/licenses/by/4.0/). I understand that any comments which I do not wish to

be included in my named report can be included as confidential comments to the editors, which will not be published.

I agree to the open peer review policy of the journal
